# Clinical and Radiological Findings of Autosomal Dominant Osteopetrosis Type II: A Case Report

**DOI:** 10.1155/2013/707343

**Published:** 2013-10-24

**Authors:** Priyanka Kant, Neelkamal Sharda, Rahul R. Bhowate

**Affiliations:** ^1^Department of Oral Medicine and Radiology, Manav Rachna Dental College, Sector 43, Aravalli Hills, Delhi-Surajkund Road, Faridabad, Haryana 121001, India; ^2^Department of Oral Medicine and Radiology, Sharad Pawar Dental College and Hospital, Sawangi, Wardha, Maharashtra 442001, India

## Abstract

Osteopetrosis is a rare inherited genetic disease characterized by sclerosis of the skeleton caused by the absence or malfunction of osteoclasts. Three distinct forms of the disease have been recognized, autosomal dominant osteopetrosis being the most common. Autosomal dominant osteopetrosis exhibits a heterogeneous trait with milder symptoms, often at later childhood or adulthood. The aim of this case report is to present the clinical and radiographic features of a 35-year-old female patient with autosomal dominant osteopetrosis type II who exhibited features of chronic generalised periodontitis, and the radiographs revealed generalised osteosclerosis and hallmark radiographic features of ADO type II, that is, “bone-within-bone appearance” and “Erlenmeyer-flask deformity.”

## 1. Introduction

The term osteopetrosis is derived from the Greek word “osteo” meaning bone and “petros” meaning stone. Osteopetrosis is referred to as “marble bone disease” and “Albers-Schönberg disease”, after the German radiologist credited with the first description of the condition in 1904 [1]. Osteopetrosis comprises a clinically and genetically heterogeneous group of conditions that share the hallmark of increased bone density on radiographs. The increase in bone density results from abnormalities in osteoclast differentiation or function [2]. In healthy bone, a steady state is achieved in which production of bone by cells called osteoblasts is balanced by bone resorption by osteoclasts. Dysfunctional osteoclasts that are observed in osteopetrosis result in bony overgrowth, leading to bones that are abnormally dense and brittle. It is believed that osteoclasts fail to release the necessary lysosomal enzymes for bone resorption into the extracellular space [3, 4]. 

Defects in different genes have been described that lead to a phenotype with osteopetrosis, and mutations in at least 10 genes have been identified as causative in humans. These defects include mutations in the gene encoding carbonic anhydrase II, the proton pump gene, and the chloride channel gene [5, 6]. Recently, the immune response has been incriminated in the pathogenesis of various metabolic bone diseases, including osteopetrosis. Both cytotoxic T lymphocyte-associated antigen 4 and programmed death-1, a newly identified immunoregulatory receptor, have been shown to negatively regulate immune responses and to affect osteoclastogenesis and bone remodeling [7]. 

 This disease has been reported in three clinical forms: (1) malignant infantile form with poor prognosis and autosomal recessive inheritance, (2) benign/adult osteopetrosis with autosomal dominant inheritance and with fewer symptoms, (3) autosomal recessive intermediate form with clinical manifestations similar to malignant form and lowest incidence rate [8–10]. 

 The overall incidence of this condition is difficult to estimate, but autosomal recessive osteopetrosis has an incidence of 1 in 250,000 births [11], and autosomal dominant osteopetrosis has an incidence of 1 in 20,000 live births [8].

 Osteopetrotic conditions vary greatly in their presentation and severity, ranging from neonatal onset with life-threatening complications such as bone marrow failure (e.g., classic or “malignant” ARO) to the incidental finding of osteopetrosis on radiographs (e.g., osteopoikilosis) [12].  Table 1 shows a summary of the key clinical manifestations, onset, severity, treatment, and prognosis of the main types of osteopetrosis [12].

 Adult osteopetrosis is the most common type of osteopetrosis; it is usually discovered later in life and has a less severe manifestation. In most patients, this pattern is inherited as an autosomal dominant trait and has been termed benign osteopetrosis. Approximately 40% of those affected are asymptomatic, and marrow failure is rare, and most of patients are diagnosed only when osteomyelitis occurs in the mandible. Other symptoms include bone pain, recurrent fractures, back pain, and degenerative arthritis [13]. Bollerslev and Andersen Jr. described two subtypes of benign autosomal dominant osteopetrosis (ADO) on the basis of radiological and clinical differences; these include ADO type I and ADO type II [8].

 ADO type I is generally very mild with a diffuse sclerosis without alterations in the bone turnover. Genetic mutations of low-density lipoprotein receptor related protein 5 gene are identified to be responsible for ADO type I thus causing an increased bone formation rather than decreased bone resorption [14, 15]. This phenotype is not associated with an increased fracture rate and is reported to be fully penetrant [8]. Radiographs reveal sclerosis of the skull, which mainly results in increased thickness of the cranial vault; however, the spine does not show much sclerosis. Cranial nerve compression is common in type I [13].

Autosomal dominant osteopetrosis type II was the first type of osteopetrosis recognized and described by Albers-Schonberg in 1904 [1]. In 75% of the cases, osteoclast chloride (CLC7) gene mutation is responsible for the clinical manifestations [12]. ADO type II is the most common form and has an extremely heterogeneous course ranging from an asymptomatic to a severe phenotype. Early death in patients with ADO type II is rare, but some patients can experience a very poor quality of life [16].

 Clinical manifestations of ADO type II include hip osteoarthritis caused by excessive toughness of the subchondral bone, facial nerve palsy, mandibular osteomyelitis caused by dental caries and abscesses, and fractures of the long-bones with or without trauma in 75% of the patients. Cranial nerve compression is rare, hearing loss and visual loss occur in fewer than 5% of the affected patients. Many patients undergo several orthopaedic procedures that are often complicated owing to the hardness and the brittleness of their skeletons. However, 20%–40% of the patients remain asymptomatic [17, 18]. 

 Skeletal alterations of patients with ADO type II are so dramatic that the diagnosis is usually easy to ascertain by standard radiographs. Radiographically, sclerosis predominates, in several sites, including the spine (vertebral end-plate thickening, or Rugger-Jersey spine), the pelvis (“bone-within-bone” structures), and the cranial base. Erlenmeyer-shaped femoral metaphysis and transverse bands of osteosclerosis are observed in the long bones. However, the radiological penetrance is only 60%–90% [12].

 Due to therarity of the disease and paucity of reported cases of autosomal dominant osteopetrosis type II in the literature, the purpose of this paper is to report a case of ADO type II in a 35-year-old female patient.

## 2. Case Description

A 35-year-old female patient reported with the chief complaint of deposits on teeth from the past 6-7 months. While recording the case history, the patient complained of intermittent mild pain in the lower back region from the past three months which aggravated on daily activity and was relieved by rest, and pain was radiating to both the lower limbs. Patient gave no history of previous fractures or swellings elsewhere in the body. There was no history of trauma and fractures in any other part of the body. 

 General examination revealed a short stature, thin built with normal skin and gait. There was pallor present in the palpebral conjunctiva, nails, and the palms. On extraoral examination hypertelorism, exophthalmos, depressed nasal bridge, broad face, and a prognathic mandible were noted. 

 Intraoral examination revealed generalised gingival inflammation, generalised shallow pockets, and gingival recession with lower anterior teeth. No caries, mobility, attrition, abrasion, and so forth were noted in any of the teeth. A provisional diagnosis of chronic generalised periodontitis was made. 

**Figure 1:**
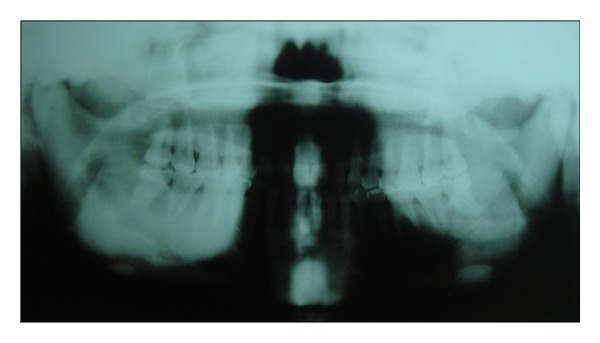
Panoramic radiograph showing signs of early periodontitis and sclerosis of the mandible and maxilla.

 Panoramic radiograph showed signs of early periodontitis with generalised mild to moderate bone loss around teeth. Generalised marked increase in the bone density, lack of distinct lamina dura, and altered trabecular pattern were noted in both jaws; the trabeculae were coarse, dense, and increased in number. Right side of the mandible showed obliteration of the inferior alveolar canal, and cotton wool appearance of the trabeculae was noted at the right angle of the mandible. Left side of the mandible was comparatively less affected, showing an increase in bone density but no cotton wool appearance and no frank obliteration of the inferior alveolar canal. The trabeculae were dense in both sides of the maxilla, the zygomatic complex appeared hyper dense on both the sides, and the maxillary sinus was hypoplastic bilaterally (Figure 1).

**Figure 2:**
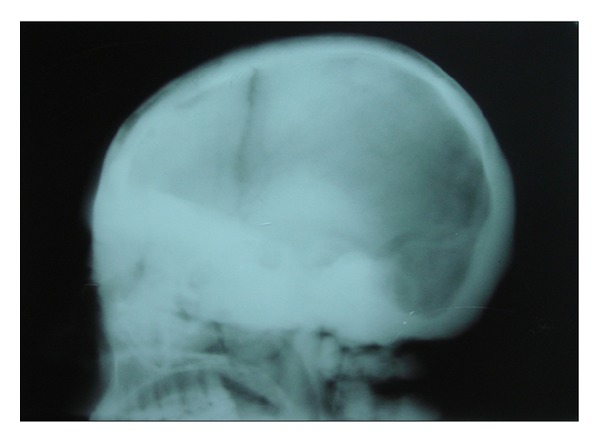
Lateral skull view showing thickening of the inner and outer cortical tables and widening of the diploic space.

**Figure 3:**
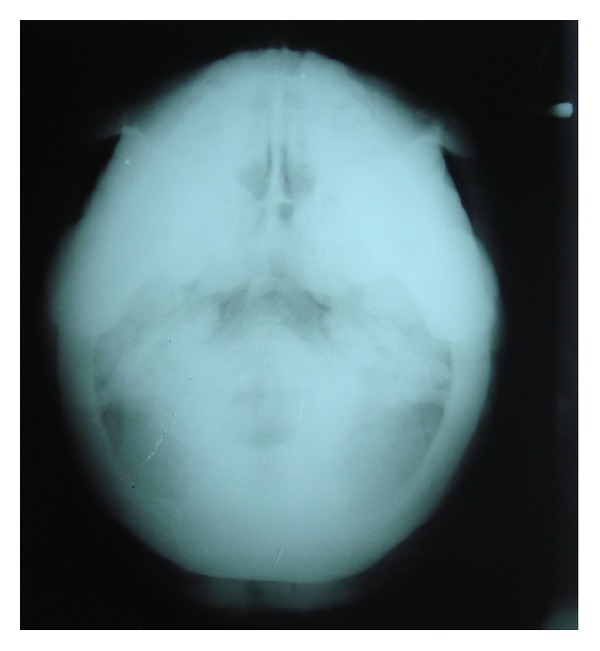
Submentovertex view showing increased radiodensity of the base of the skull, hypoplasia of foramen magnum, and obliteration of other foramina.

**Figure 4:**
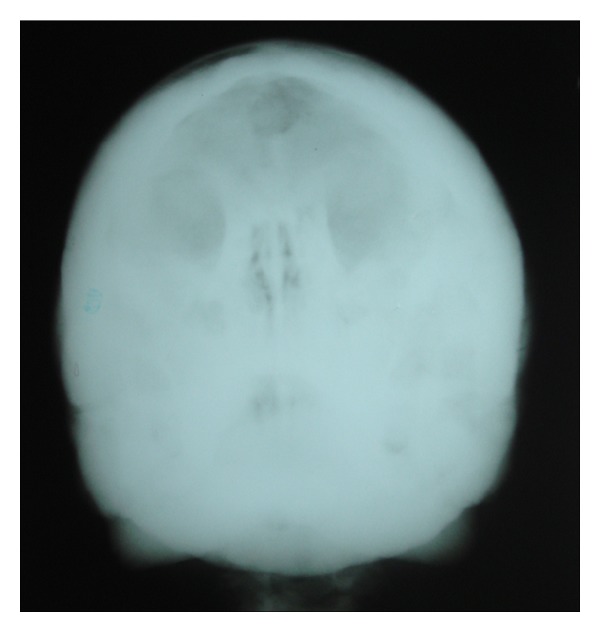
PNS view showing osteosclerosis and hypoplasia of sinuses and the nasal cavity.

**Figure 5:**
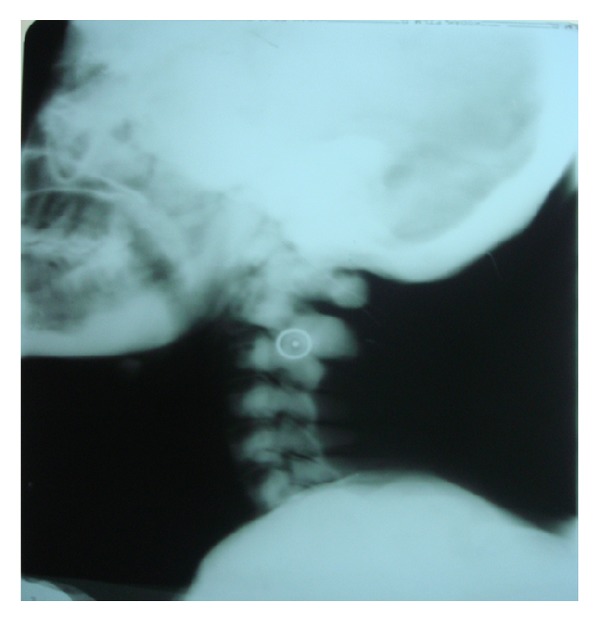
Anteroposterior view of the cervical vertebra showing increased radiodensity in all the cervical vertebrae.

 After noting these features, the patient was subjected to further radiographic examination to study the skull, paranasal sinuses and the cervical spine (PNS, submentovertex, lateral skull, and cervical spine) which revealed increased bone density of all the bones of the skull, face, and the cervical spine. These views revealed thickening of the cortical boundaries of all the bones with obliteration of the marrow spaces. Lateral skull view revealed thickening of the inner and outer cortical tables and widening of the diploic space (Figure 2). The base of the skull appeared highly radiodense with loss of trabecular pattern, and there was hypoplasia of foramen magnum, and other foramina were obliterated (Figure 3). The nasal cavity and the sinuses were also hypoplastic showing high areas of radiodensity (Figure 4). Anteroposterior view of cervical spine showed increased radiodensity in all the cervical vertebrae (Figure 5).

**Figure 6:**
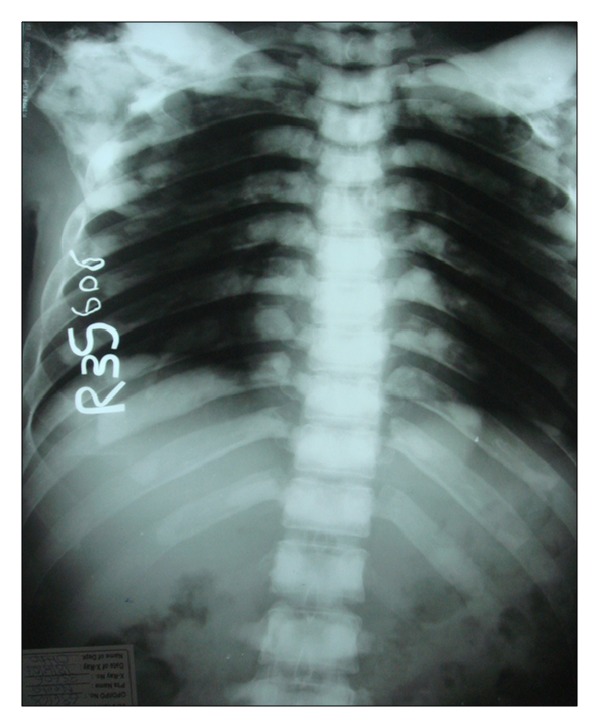
PA chest showing generalised osteosclerosis in the thoracic cage and both clavicular bones.

**Figure 7:**
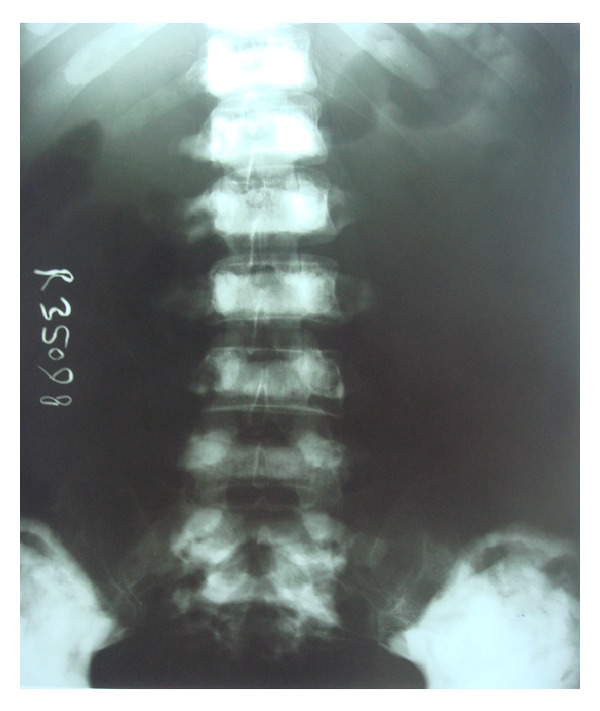
Anteroposterior view lumbar spine showing sclerosis at all the levels and “bone-in-bone appearance.”

**Figure 8:**
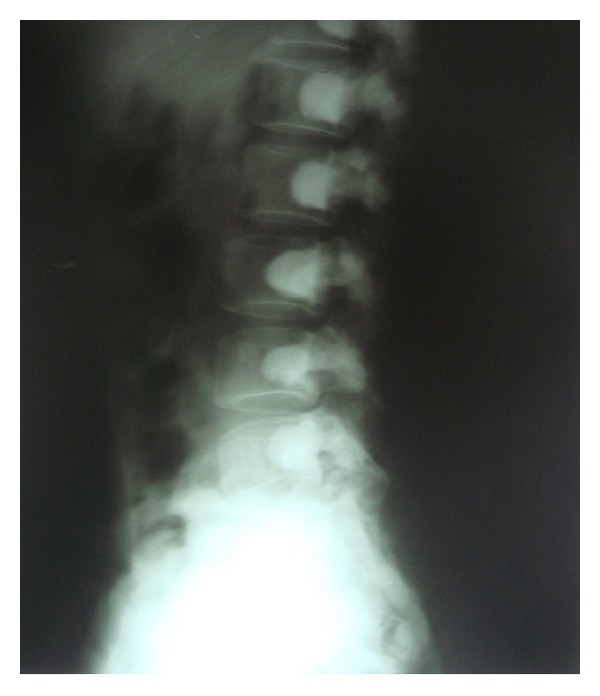
Lateral view of the lumbar spine showing “bone-within-bone appearance.”

**Figure 9:**
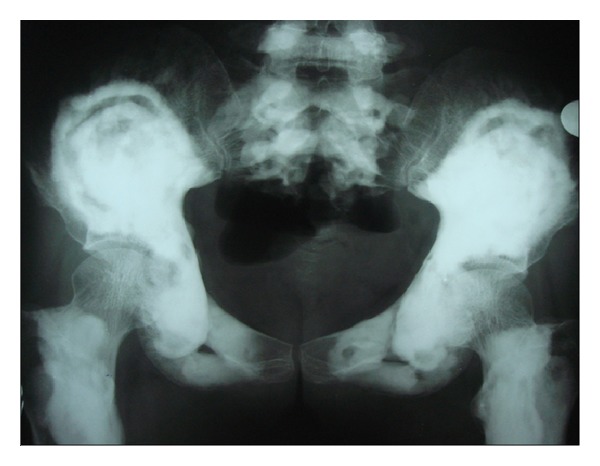
Anteroposterior view of the pelvis showing expanding osteosclerosis of the pelvic bone and the iliac wings.

**Figure 10:**
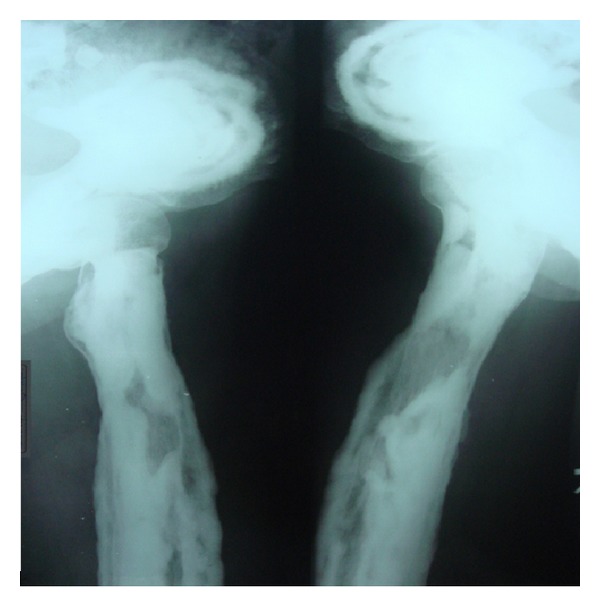
Anteroposterior view of the hip joint showed generalised sclerosis of the pelvic rami and the femur bone.

**Figure 11:**
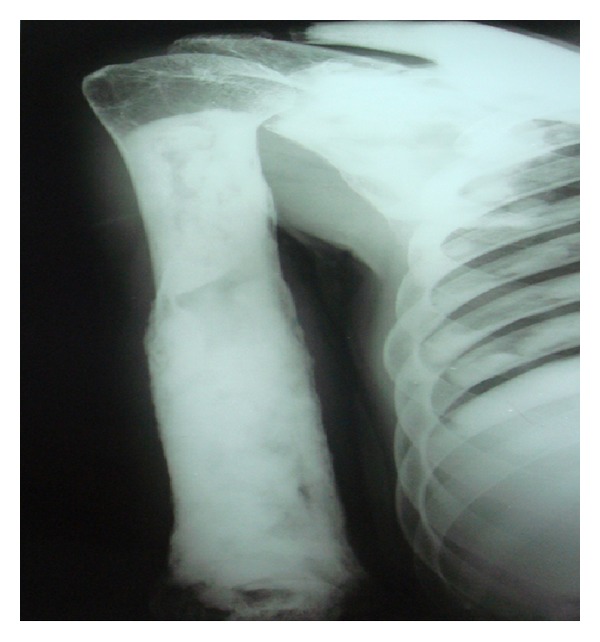
Radiograph of the humerus showing osteosclerosis of the humerus and scapula and typical “funnel-like appearance” (Erlenmeyer-flask deformity) in the humerus.

**Figure 12:**
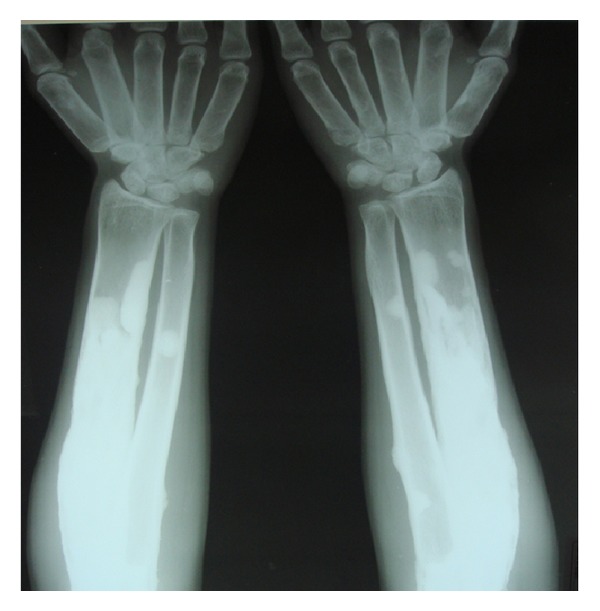
Anteroposterior view of radius and ulna showing increased radiodensity in all the bones, smoothening of the bone surfaces, and cylindrical appearance of the metacarpals.

 Following these findings, the patient was subjected to whole body radiographic survey to rule out the presence of pathological fractures in the bones. Posteroanterior chest radiograph showed a generalised increase in the bone density throughout the thoracic cage and both clavicular bones with normal heart shadow and normal lung fields (Figure 6). Radiograph of the lumbar spine AP and lateral view showed sclerosis at all the levels and “bone-in-bone appearance.” Normal spinous processes, normal height, and morphology of the individual vertebrae were noted (Figures 7 and 8). Radiograph of the pelvis showed expanding osteosclerosis of the pelvic bone and the iliac wings (Figure 9). Radiograph of the hip joint showed generalised sclerosis of the pelvic rami and the femur bone (Figure 10). Radiograph of the humerus showing dense sclerosis of the humerus and scapula and “funnel-like appearance” in the humerus (Figure 11). AP view of radius and ulna showed increased radiodensity in all the bones, smoothening of the bone surfaces, and cylindrical metacarpals (Figure 12).

 Hematologic investigations showed Hb of 10.8 g/dL, white blood cells 6.5 k/uL, and platelet count 130 k/uL, and ESR was in the normal range. Red cell indices and iron profile were normal. Peripheral smear showed normocytic and normochromic anemia. Renal function and serum electrolytes were within normal limits (creatinine 0.61 mg/dL, sodium 136 mEq/L, and potassium 3.9 mEq/L). Liver function tests were normal (serum bilirubin 0.75 mg/dL, SGOT 20 IU/L, SGPT 18.3 IU/L, alkaline phosphatase 80 IU/L, total protein 7 gms/L, albumin 4.6 gms/L, globulin 3 gms/L), and serum calcium was 8.9 mg/dL, and serum phosphorus was 3.0 mg/dL.

**Figure 13:**
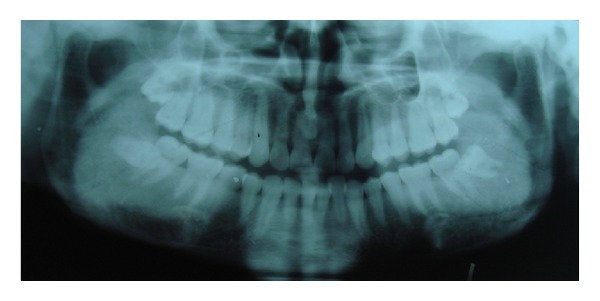
Panoramic radiograph of the patient's brother.

 Family history of the patient was unclear. Panoramic radiograph of the patient's brother was also performed, which was normal (Figure 13).

 Considering the history, along with clinical, radiological, and laboratory findings and orthopaedician's opinion, a final diagnosis of autosomal dominant osteopetrosis type II was given. 

 Supragingival, subgingival scaling, and root planning were done for the patient. Patient was also advised to rinse her mouth with 0.2% chlorhexidine gluconate mouth wash twice daily, and topical fluoride application was done. To combat lower back pain, patient was prescribed a topical muscle relaxant and analgesic for SOS use. On regular follow-up visits for 6 months, the patient showed no evidence of fractures and osteomyelitis affecting any bone, no signs of cranial nerve involvement and no dental caries and abscesses. The patient also maintained a good oral hygiene. The patient was lost to follow up after 6 months. 

## 3. Discussion

ADO type II is caused by mutation in gene encoding chloride channel 7. Del Fattore et al. reported ClCN7 mutation in 77.8% of the patients [19]. Cleiren et al. reported 7 distinctive mutations in ClCN7 gene in 12 unrelated families with ADO type II [20]. 

ADO type II is the most common form of osteopetrosis with an estimated prevalence of 1 in 20,000 births. Age of onset of ADO type II is late childhood or adolescence [12]. Del Fattore et al. studied 20 patients with type II autosomal dominant osteopetrosis, the youngest patient being 3 years old and the oldest patient being 63 years old [19]. Waguespack et al. discussed 62 cases with ADO type II, among these 19 patients were ≤18 years of age and 43 patients were >18 years of age [21].

 The incidence of autosomal dominant osteopetrosis type II is reported to be the same for males and females. However, 55% of the patients reported by Del Fattore et al. were males and 45% of the patients were females [19]. Waguespack et al. reported a male predominance in their study too [21]. Our patient was a 35-year-old female.

Most individuals diagnosed with autosomal dominant osteopetrosis type II have an affected parent, although in our case the family history was unclear, and a panoramic radiograph of the patient's brother was performed which was normal. 

ADO type II has an extremely heterogeneous course ranging from an asymptomatic to a severe phenotype [22]. Clinical manifestations of ADO type II are dominated by long-bone fractures [19]. Other classic manifestations of ADO type II include hip osteoarthritis, scoliosis, and osteomyelitis, particularly affecting the mandible. Cranial nerve compression is a rare complication [23].

Clinical manifestation in the form of diffuse pain was reported in 30% of the patients by Del Fattore et al. with ADO type II and fractures in ≤3 bones was reported in 20% of the patients. 10% of the patients reported fractures in 4–10 bones, and more than 10 bones were fractured in 15% of the patients [19]. Fracture was the most prevalent clinical manifestations occurring in 84% of all ADO type II subjects reported by Waguespack et al., fractures of the pelvis, hip, and femur being most the common (84%) [21]. Clinical manifestations were experienced in 81% of the patients with ADO type II as reported by Bénichou et al. and fractures of femur and ribs being the most common (76%) [16]. El-Tawil and Stoker reported a fracture rate of 62% (femur most common) [24]. 67% fractures most commonly in the appendicular skeleton was reported by Bollerslev and Andersen Jr. [25]. In our case, the patient reported mild pain in the lower back region which was radiating to both the lower limbs, but there was no history of fractures reported in any of the bones, and full body radiographic survey of our patient showed no evidence of fracture in any of the other bones.

 Visual impairment and central nervous system involvement is rare in autosomal dominant osteopetrosis type II [12]. Severe visual loss was reported in 19% of the patients by Waguespack et al. [21]. Bénichou et al. reported visual loss in 5% of the patients [16]. There were no signs of visual loss and central nervous system involvement in our case.

 Osteomyelitis is the common clinical manifestation in patients with autosomal dominant osteopetrosis type II. Waguespack et al. reported osteomyelitis of the femur in 2 individuals after surgical repair of the fracture [21]. Bénichou et al. [16] reported osteomyelitis in 11% of the patients. Our case had no signs and symptoms of osteomyelitis affecting any of the bones.

 The dental changes reported to be associated with osteopetrosis include disturbance of tooth eruption, hypodontia, malformed teeth, multiple caries, enamel dysplasia, abnormal pulp chambers, and hypercementosis [12, 13]. The main complication in patients with autosomal dominant osteopetrosis type II is osteomyelitis, particularly affecting the mandible in association with dental caries and abscess [12]. Waguespack et al. reported osteomyelitis affecting either the maxilla and or mandible in 13% of the patients [21]. Our case showed no abnormalities associated with the teeth, and there was no evidence of osteomyelitis affecting either the maxilla or mandible, but in our case there was early periodontitis with generalised gingival inflammation, shallow pockets, and gingival recession with lower anteriors.

 Dental radiographs of patients with ADO type II reveal a generalized increase in radio density of the maxilla and mandible with abnormal trabecular pattern and diminished marrow spaces, constriction of the inferior alveolar nerve canal, and dental pulp canal as well as thickening of the lamina dura. Maxillary sinuses also appear hypoplastic [26]. Panoramic radiograph of our case showed generalised increase in the bone density, lack of distinct lamina dura, and absence of normal trabecular pattern involving both jaws, obliteration of the inferior alveolar nerve canal, cotton wool appearance on the right side of the mandible, and bilateral hypoplasia of the maxillary sinus.

 In radiological manifestations of ADO type II, osteosclerosis of the spine predominates, with a “sandwich vertebra” appearance. Most individuals have a “bone-within-bone” appearance primarily in the iliac wings, but also in other bones. Transverse bands of sclerosis, perpendicular to the main axis, are often observed in long bones. Increase in the skull base density can also be seen [16]. Bollerslev and Andersen Jr. reported striking radiographic findings like diffuse systemic osteosclerosis, “rugger jersey spine,” and “bone-within-bone appearance” in the pelvis in 15 patients autosomal dominant osteopetrosis type II patients, and the cranial vault was unaffected in almost all the patients [25]. Del Fattore et al. reported generalised osteosclerosis in 43% of the cases, rugger jersey spine was noted in 52.3% of the patients, and 33.3% patients had bone-in-bone appearance [19]. Our patient had hallmark diffuse sclerosis, affecting the skull, spine, pelvis, and appendicular bones; bone modelling defects at the metaphyses of long bones, such as funnel-like appearance (“Erlenmeyer-flask” deformity), and characteristic lucent bands; “bone-within-bone” appearance in the vertebrae.

The differential diagnosis that can be considered includes other sclerosing bone dysplasias, such as pycnodysostosis, craniometaphyseal dysplasia, diaphyseal dysplasia, melorheostosis, osteopoikilosis, and osteopathia striata. Fluoride poisoning and secondary hyperparathyroidism from renal osteodystrophy also may produce a diffuse osteosclerosis [4].

 Bone marrow function in benign osteopetrosis is not compromised, and the hematological findings are often normal [12]. Waguespack et al. reported significant bone marrow failure necessitating haematological supportive care in 3% of the patients with ADO type II [21]. However, in our patient hemoglobin was 10.8 g/dL, and white blood cell count and platelet count were in the normal range. Peripheral smear showed normocytic normochromic anemia. Other investigations, that is, renal function test, liver function test, ESR, serum calcium, and serum phosphorus, were also within the normal limit.

 The diagnosis of osteopetrosis is based on radiological and clinical features and these findings are sufficiently characteristic to make a definite diagnosis, and there is no need to perform a genetic study to confirm the disease. Furthermore, biopsy must be avoided because of a marked infection risk [26, 27].

 At present, no effective medical treatment for osteopetrosis exists. Treatment is largely supportive and is aimed at providing multidisciplinary surveillance and symptomatic management of complications. Fractures and arthritis are common and require treatment by an experienced orthopaedic surgeon due to the brittleness of the bones, and the relatively frequent occurrence of secondary complications such as delayed union or nonunion of fractures and osteomyelitis [12].

 Therefore, routine dental surveillance and maintenance of oral hygiene form an integral part of management and play an important role in preventing more severe complications such as osteomyelitis of the mandible [12]. Moreover, teeth should be endodontically treated, if possible, rather than extracted, due to the increased risk of infection. If osteomyelitis occurs, surgical intervention must be considered because antibiotics do not reach the compromised region [17].

 In our case scaling, root planning, and topical fluoride application were done, and the patient was instructed to maintain good oral hygiene. The patient was also advised to rinse her mouth with 0.2% chlorhexidine gluconate mouth wash. The patient was prescribed Volini gel (diclofenac diethylamine 1.16%, methyl salicylate 10%, and linseed oil 3%) for topical application twice daily on the lower back. The patient was followed for 6 months, and during this period the patient showed no evidence of fractures and osteomyelitis affecting any bone. No signs of cranial nerve involvement, dental caries, and abscesses were noted. Patient was lost to follow up after 6 months.

## 4. Conclusion 

Benign osteopetrosis is a rare disorder, which might be characterized by an asymptomatic clinical picture. Therefore, a proper clinical and radiographic investigation is essential for accurate diagnosis. Dental problems like delayed tooth eruption, ankylosis, abscesses, cysts, and fistulas are common in patients with benign osteopetrosis. Constriction of the inferior alveolar nerve canal and dental pulp canal and thickening of the lamina dura are routinely seen in dental radiographs because of increased bone density. Therefore, dental practitioners can play an important role in early diagnosis and treatment planning of the patients with benign osteopetrosis. Moreover, because of the high infection risk and increased susceptibility to jaw fracture in these patients, they should receive increased attention and prophylactic dental treatment to maintain their fragile oral health status. Continuous and vigorous preventive measures should be performed for patients to prevent any complications.

## Figures and Tables

**Table 1:** Summary of the key clinical manifestations, onset, severity, treatment, prognosis, and recurrence risks of the main types of osteopetrosis.

	Autosomal recessive osteopetrosis (ARO)					
Osteopetrosis subtype				X-linked osteopetrosis, lymphedema, anhidrotic ectodermal dysplasia, and immunodeficiency (OLEDAID)	Intermediate osteopetrosis (IRO)	Autosomal dominant osteopetrosis (Alber's Schönberg disease)
	Classic	Neuropathic	ARO with RTA			
Genetic basis	TCIRG	CLCN7, OSTM1	Carbonic anhydrase II	IKBKG (NEMO)	CLCN7, PLEKHM1	CLCN7

Skeletal manifestations	Increased bone density, diffuse and focal sclerosis of varying severity Modelling defects at metaphyses Pathologic fractures Osteomyelitis Dental abnormalities: tooth eruption defects and dental caries					

Other manifestations	Pancytopenia, extramedullary hematopoiesis, hepatosplenomegaly, cranial nerve compression (II, VII, and VIII), hydrocephalus, and hypocalcemia	As for classic ARO, but primary neurodegeneration, including retinal atrophy	Renal tubular acidosis, developmental delay, intracranial calcification, cranial nerve compression, and rare bone marrow impairement	Anhidrotic ectodermal dysplasia, lymphedema, and immunodeficiency resulting in overwhelming infection	Anaemia and extramedullary hematopoiesis, occasional optic nerve compression	Moderate haematological failure, cranial nerve compression

Onset	Perinatal	Perinatal	Infancy	Infancy	Childhood	Late childhood or adolescence

Severity	Severe	Severe	Moderate	Severe	Mild to moderate	Mild to moderate, occasionally severe

Treatment	Supportive HSCT	Supportive	Supportive May benefit from HSCT	Supportive	Supportive	Supportive

Prognosis	Poor Fatal in Infancy	Poor Fatal in Infancy	Variable	Poor Fatal in early childhood	Variable	Normal life expectancy
